# AZT as a telomerase inhibitor

**DOI:** 10.3389/fonc.2012.00113

**Published:** 2012-09-06

**Authors:** Daniel E. Gomez, Romina G. Armando, Daniel F. Alonso

**Affiliations:** Laboratory of Molecular Oncology, Department of Science and Technology, Quilmes National University, BernalBuenos Aires, Argentina

**Keywords:** telomere, telomerase, inhibitors, AZT

## Abstract

Telomerase is a highly specialized reverse transcriptase (RT) and the maintenance of telomeric length is determined by this specific enzyme. The human holoenzyme telomerase is a ribonucleoprotein composed by a catalytic subunit, hTERT, an RNA component, hTR, and a group of associated proteins. Telomerase is normally expressed in embryonic cells and is repressed during adulthood. The enzyme is reexpressed in around 85% of solid tumors. This observation makes it a potential target for developing drugs that could be developed for therapeutic purposes. The identification of the hTERT as a functional catalytic RT prompted studies of inhibiting telomerase with the HIV RT inhibitor azidothymidine (AZT). Previously, we have demonstrated that AZT binds preferentially to telomeres, inhibits telomerase and enhances tumor cell senescence, and apoptosis after AZT treatment in breast mammary adenocarcinoma cells. Since then, several studies have considered AZT for telomerase inhibition and have led to potential clinical strategies for anticancer therapy. This review covers present thinking of the inhibition of telomerase by AZT and future treatment protocols using the drug.

## INTRODUCTION

Telomerase is a ribonucleoprotein that elongates the telomeres in eukaryotic cells. The enzyme is composed of a catalytic subunit, hTERT, a RNA component, hTR, and a group of associated proteins. Based on experiments in fibroblasts, in the absence of telomerase, cellular telomeres are seen to shorten with an increasing number of cell divisions. It was suggested that the lost of telomeres could account for cell senescence after a given number of duplications (Harley et al., 1991).

Studies on fibroblasts cultures have indicated that telomeric length could be used to predict the replicative capacity of these cells (Allsoop and Harley, 1995). It was proposed that the lost of telomeric repeats, when a critic telomeric length was reached, induced a signal that influences the cell cycle process and replicative senescence (Campisi, 1997). According to this model, telomeres act as a mitotic clock that determines the replicative life of a cell (Vaziri and Benchimol, 1998). The use of the telomeric length as a marker of the cells’ replicative history has been also described by Chang and Harley (1995) who demonstrated that telomeric length decreases in function of the passages of endothelial cells in culture. Later, Bodnar et al. (1996) showed that the reintroduction of the catalytic compound of telomerase in primary human fibroblasts and endothelial cells that lack telomerase activity elongated the telomeric repetitions resulting in significant increases in replicative cell life.

Telomerase is active in the germ line and stem cells but is inactive in most somatic cells. However, telomerase activity is found in most immortalized cell lines and in 85–90% of human tumors. It was also reported that telomerase activity was present in 90 of 101 biopsies that represented 12 human tumor types, but none in 50 normal somatic tissues (Dhaeene et al., 2000).

**FIGURE 1:**
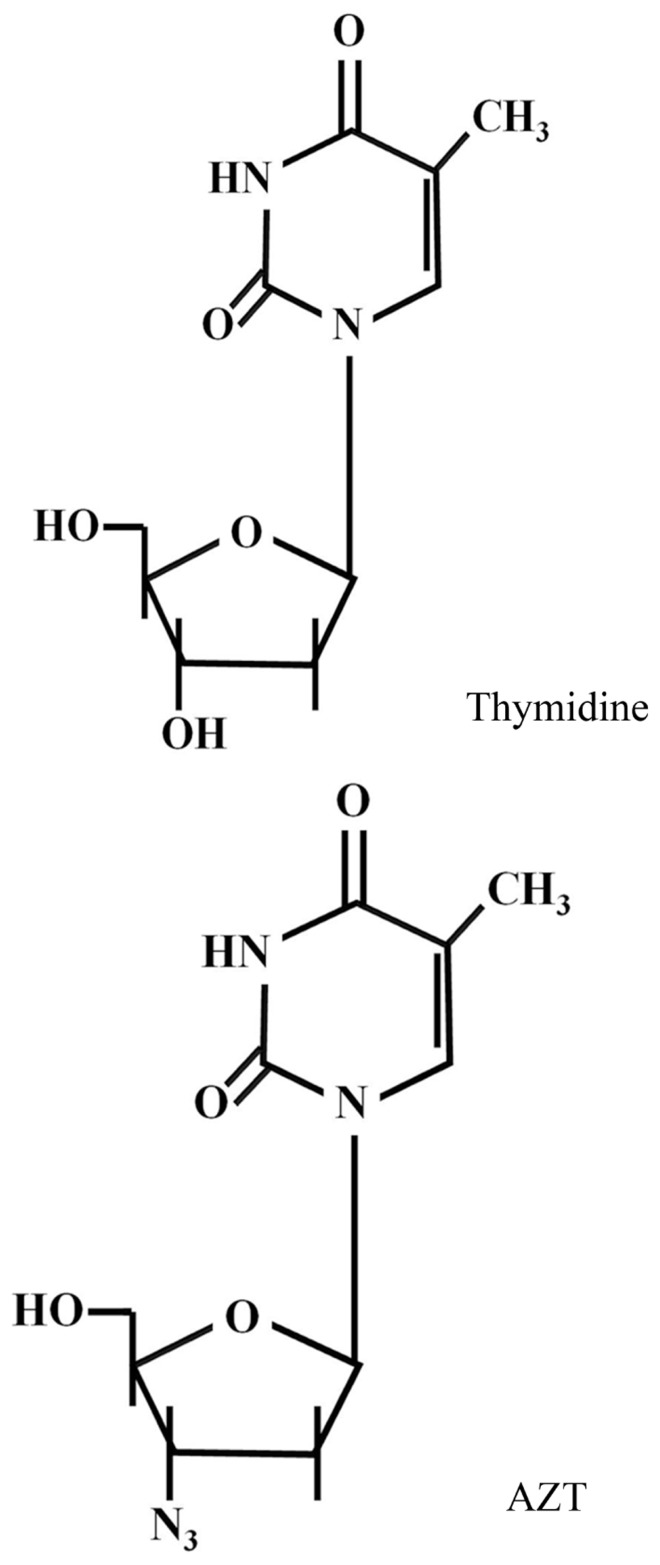
**AZT structure**.

3′-Azido-2′,3′-dideoxythymidine [azidothymidine (AZT) or zidovudine], is a thymidine analog (**Figure 1**) used in the treatment of AIDS, since it blocks the replication of human immunodeficiency virus-1 (HIV-1) by competitively inhibiting reverse transcriptase (RT). It is phosphorylated intracellularly to AZT-triphosphate (AZT-TP) by thymidine kinase, and is finally incorporated into viral DNA blocking chain elongation by RT (Falchetti et al., 2005). AZT-TP can also be incorporated into eukaryotic DNA in place of thymidine, although it has low affinity for DNA polymerases α, β, and γ and high affinity for RT (Faraj et al., 2000). The identification of the hTERT component of telomerase as a functional catalytic RT, structurally similar to HIV RT, prompted studies about the feasibility of inhibiting telomerase with known RT viral inhibitors, such as AZT (Hájec et al., 2005).

## AZT INHIBITION

Strahl and Blackburn (1994) demonstrated in *Tetrahymena thermophila* that AZT shortened its telomeres by decreasing the *de novo* telomere addition. In the same year it was demonstrated that there are strong links between telomerase activity and cancer (Kim et al., 1994). These two pioneering papers led to the speculation that AZT could be acting upon the telomere/telomerase complex in cancer cells.

In spite of the low affinity of AZT for mammalian DNA polymerases, the drug can be incorporated into eukaryotic DNA. Furthermore it was demonstrated that AZT was preferentially integrated into the telomeric region of CHO DNA using immunofluorescence labeled antibodies against AZT (Olivero and Poirier, 1993). In 1995, we developed a methodology to quantitate the preferential incorporation of AZT comparing the amount of [^3^H]-AZT bound to telomeric and non-telomeric sequences of CHO cell DNA (Gomez et al., 1995).

Briefly, telomeric DNA was separated from genomic DNA by digesting with restriction enzymes and size fractionation. We quantitatively compared the amount of [^3^H]-AZT bound to telomeric and non-telomeric sequences of CHO cell DNA. DNA from cells exposed to 800 μM of [^3^H]-AZT for 24 h was digested with a mixture of restriction enzymes, frequent cutters in the overall genome, without restriction sites in the telomeric repeat (*Sau*3AI, *Alu*I, and *Rsa*I). The digest was then loaded into Chroma Spin-1000 columns to isolate fragments of 1 kb and upward. The first fraction to be eluted was characterized as the telomeric fraction (TF) whereas the non-telomeric fraction (NTF) eluted later in second place. TF was comprised of longer sequences (>2 kb) compared to NTF. This was confirmed by a Southern blot using a human telomeric probe. Radioactivity associated with each fraction revealed a threefold increase in [^3^H]-AZT incorporated in the TF compared to NTF. No preferential telomeric binding was detected for [^3^H]-thymidine (Tdr) or [H-3]-5′-bromodeoxyuridine (BrdU) in similar experiments. The failure of thymidine or BrdU to preferentially incorporate into telomeric repeats supported the hypothesis that differential incorporation of AZT into TF was not a direct result of the telomeric sequence itself but most probably a phenomenon related to the specificity of a polymerase such as telomerase. Primary cultures of mouse dermal fibroblasts, cells with large telomeric repeats that lack telomerase, did not preferentially incorporate AZT into the TF. Bal 31 digested DNA consists of mononucleotides, including AZT, from both 3′ termini of the two strands of linear DNA. When the chromosomal ends of high molecular weight [^3^H]-AZT-DNA were digested with Bal 31, the radioactivity was double in the TF (short-term digest) compared with the NTF (longer digestions). Therefore incorporation of AZT in CHO immortalized cells but not in primary fibroblasts (that lack telomerase) indirectly showed that AZT incorporation could be telomerase-mediated.

Later, using different experimental models different groups demonstrated that AZT inhibited telomerase and/or reduce telomerase length. Strahl and Blackburn (1996) showed the effect of AZT on the telomere length and cell growth properties of two immortalized human lymphoid cell lines, the B cell line JY616 and the T cell line Jurkat E6-1. They found that passaging in the presence of 100 μM AZT caused marked progressive telomere shortening over several weeks in some but not all T and B cell cultures, without changing cell growth rates. Telomerase activity was present in both cell lines and was inhibited *in vitro* by AZT (Strahl and Blackburn, 1996). At the same time, Yegorov et al. (1996) reported that the spontaneous transformation of mouse embryonic fibroblasts in the presence of AZT led to the formation of telomerase-free clones after 3 months treatment. AZT induced senescence-like processes in cultures of immortal mouse fibroblasts. After long-term incubations, cell proliferation gradually decreased, their morphology becoming similar to that of the senescent cells. The process was reversible: after inhibitor removal, the cells entered mitoses. One or two weeks after AZT removal large mitotic cells were observed. Moreover, standard karyotyping revealed that these cells were polyploid. It was concluded that AZT block telomerase function in mouse cells (Yegorov et al., 1996). Later, Olivero et al. (1997) studied the transplacental effects of AZT in mice and monkeys founding shorter chromosomal telomeres in liver and brain tissues from most AZT-exposed newborn mice. Melana et al. (1998) investigated the effect of AZT in four breast cancer cell lines, T4 leukemia, and a normal breast cell line *in vitro*. AZT inhibited the growth of all tumoral cell lines, covering a wide range of concentrations. The IC_50_ varied from 0.25 to 1.35 mM for the cancer cell lines and no correlation of IC_50_ with the rate of cell growth was noted. Variations in IC_50_ may reflect differences in absorption, excretion, and/or phosphorylation of AZT between the cell lines. Furthermore, they found that AZT inhibited colony formation in soft agar and telomerase activity (Melana et al., 1998). Similarly we showed for the first time, that telomere shortening by AZT was an irreversible process, suggesting that telomerase activity was possible after removal of the drug from the culture medium (Gomez et al., 1998).

In these experiments we subjected HeLa cells to long-term exposure with AZT to evaluate telomere shortening and if so, the reversibility of the phenomenon. The shortening of the telomeric sequences of HeLa cells cultured with 800 μM AZT was observed after 15 passages. Southern blots of HeLa DNA cultured with AZT and digested with *Sau*3AI, *Alu*I, and *Rsa*I revealed a progressive shortening of the telomeric repeats when probed with a human biotinylated telomeric probe. The shortening appeared to be linear with time in culture and was irreversible, because the shortened telomeric repeats were not elongated after culturing in the absence of AZT for 25 additional passages. Fluorescence *in situ* hybridization was also performed in cells growing with or without AZT. Bright spots representing hybridization of fluorescent telomeric probes to the telomeres were noted and weaker signals were observed in cells exposed to AZT as compared to untreated cells. No evidence of senescence could be detected. Markers of senescence such as p21 (Waf1), small proline rich proteins (spr), and apoptosis, were analyzed after AZT treatment. The results were indistinguishable from the control untreated cells or the recovered cells with no morphological or biochemical changes attributable to senescence being observed. The fact that telomere length was decreased in HeLa cells after long-term exposure to AZT without any evidence of senescence could be the result of different mechanisms. Firstly, the number of AZT-treated passages could be insufficient for a senescence program to be triggered, and secondly telomeres shortening to a critical length could induce a compensatory mechanism of preservation so preventing further losses, known as alternative lengthening of telomeres or ALT (Gan et al., 2002). Thirdly, an AZT-resistant phenotype could have developed as a result of selection following the treatment.

More recently evidence supporting the above view has been presented, for example, Multani et al. (1998) using fluorescence *in situ* hybridization found a reduction of telomeric signals in murine melanoma (K-1735 clone X-21) and human breast cancer cell line (MCF-7) treated with AZT. Similar results were observed in human endometrial carcinoma cells (HEC-1) whereas inhibition of telomerase activity and telomere length were seen to be dependent on the AZT concentration and duration of exposure. The same authors also found that in HEC-1 cells the effects of cisplatin were enhanced with a marked reduction in cell proliferation, appearance of morphological changes and senescent-like cells in the presence of AZT (Murakami et al., 1999). Similarly, synergistic interactions between paclitaxel and AZT (Johnston et al., 2003) and between AZT and 5-fluorouracil (Brown et al., 2003) were described. Beltz et al. (1999) evaluated the effectiveness of AZT in leukemic cell lines and blood lymphocytes founding in the presence of the drug that cell growth was decreased and that AZT inhibition was manifested at 48–96 h after addition to the cultures. The inhibition was partially to totally reversible upon inhibitor removal, indicating that AZT was not cytotoxic but only suppressed cell growth temporarily (Beltz et al., 1999). Similarly, Haïk et al. (2000) found that AZT blocked in a dose-dependant manner the FGF2-induced proliferation of neural precursor cells.

In 2001 we investigated the effects of chronic *in vitro* AZT exposure on F3II mouse mammary carcinoma cells. Treatment with 800 μM AZT for at least 30 passages completely inhibited telomerase activity on F3II mammary carcinoma cells. We demonstrated, for the first time, that AZT-treated tumor cells have a reduced tumorigenicity in syngeneic BALB/c mice. Tumor incidence was reduced and survival was prolonged in animals inoculated with AZT-treated cells compared to control. The number and size of spontaneous metastases were also decreased in animals inoculated with AZT-treated cells. In addition, we presented morphological and biochemical evidence indicating senescence. At passage 34, F3II cells acquired the rounded and enlarged morphology characteristic of senescent cells. Histochemical staining with the senescence biomarker SA-β-galactosidase was positive in AZT-treated cells. To evaluate whether replicative senescence produced by long-term AZT treatment leads to apoptosis, caspase-3 activity was measured and found to significantly increase in AZT-treated tumor cells with 2.6-fold greater activity compared to control cells after more than 50 *in vitro* passages in the presence of the compound. In a different set of experiments, animals receiving 2 × 10^5^ tumor cells were sacrificed on day 60, and s.c. tumors and lungs were evaluated. The mitotic index was similar in both control and treated groups. Accordingly, there was no difference in PCNA expression. However, the apoptotic index was dramatically increased in tumors originated from AZT-treated cells. Our observations provided direct evidence that chronic AZT treatment may be sufficient to reverse tumor cell immortality and cause a senescent and apoptotic phenotype (Tejera et al., 2001).

Similar results were found using other tumor lines including parathyroid cancer cells (Falchetti et al., 2005) and hepatocellular carcinoma in rats (Jeng et al., 2011). In respect to apoptosis, Ji et al. (2005) described that the telomerase subunits, hTERT and c-Myc were the first to show reduced activity after AZT treatment, followed by changes in hTR, Mad1, and hTEP1. Later, Fang and Beland (2009) using a hepatoma cell line (HepG2) indicated that AZT caused a decrease in checkpoint kinase 1 (Chk1) and kinase 2 (Chk2) and an increase in phosphorylated Chk1 (Ser345) and Chk2 (Thr68).

## CONCLUSION

Due to the structural and mechanistic similarities between telomerase and HIV-1 RT, it has been postulated that known RT inhibitors may potentially inhibit human telomerase. The experimentational results outlined here support this view. However, doses and time of treatment are varied and the precise mechanism of AZT action still remains elusive. AZT by itself could produce a specific inhibition of telomerase by other means, independently of its action as a chain terminator. For instance, p53 seems to have a role in telomerase inhibition by AZT but more studies are required to strengthen this view (Datta et al., 2006). Lately, a number of non-telomeric telomerase functions as transcriptional modulation of the Wnt-β-catenin signaling pathway and RNA-dependent RNA polymerase activity have been described but so far they have not been conclusively linked to providing antitumoral effects. More experimental work is required to see its potential role in tumor biology.

Two main considerations should be taken into account regarding the antitumorigenic potential of the drug. First, as other chemotherapeutic agents, AZT appears to have, under certain conditions, potential tumorigenic properties (Olivero et al., 1997). Second, as the shortening of telomeres is a slow process, the dynamics of the disease could put at risk the life of the patient before the action of AZT is fully functional.

The most appealing criticism (which is still purely speculative) is the clinical relevance of inhibiting telomerase in cancer patients. According to the paradigm currently proposed for telomeres and telomerases, it can be predicted that telomerase inhibition will not affect a tumor until its telomeres reach the critical size for entering senescence. This means that during anti-telomerase therapy, the tumor cells will continue to grow undergoing 20–30 divisions until the telomeres reach a critical size leading to tumor senescence. Therefore, concerns must be expressed when attempting to treat advanced tumors with AZT (Lavelle et al., 2000). However, this mode of treatment could constitute a good adjuvant therapy in cases where conventional treatments reduce the bulk of tumor mass giving time for AZT to act in attacking the remnant surviving tumor cells.

Azidothymidine is used to treat several virus-associated human cancers, including AIDS-related Kaposi sarcoma, Kaposi sarcoma-associated primary effusion lymphoma, Epstein–Barr-associated lymphoma, primary central nervous system lymphoma, and adult T cell leukemia (Datta et al., 2006). As shown in **Table 1** in non-viral tumors, AZT has been used in phase I and II clinical trials alone or in combination with other drugs for gastrointestinal cancers, pancreatic cancer, and other advanced malignancies with some tumor regression being reported (Posner et al., 1992; Marchbanks et al., 1995; Clark et al., 1996; Miller et al., 1996; Falcone et al., 1997). Although extremely promising, more clinical trials using AZT are needed to understand the full potential of this agent in a clinical setting.

**Table 1:** Cancer clinical trials using AZT.

Scheme	Reference	Notes
AZT	Marchbanks et al. (1995)	Phase I in patients with solid tumors
AZT + methotrexate	Miller et al. (1996)	Phase II in advanced adenocarcinoma of pancreas and hepatocellular carcinoma
AZT + *cis*-diamminedichloroplatinum	Morgan et al. (2003)	Phase I in patients with advanced malignancies
AZT + 5-fluorouracil + leucovorin	Posner et al. (1992)	Phase I in advanced malignant tumors
AZT + 5-fluorouracil + leucovorin	Clark et al. (1996)	Phase II in metastatic colorectal cancer
AZT + 5-fluorouracil + leucovorin	Falcone et al. (1997)	Phase II in metastatic colorectal cancer

## Conflict of Interest Statement

The authors declare that the research was conducted in the absence of any commercial or financial relationships that could be construed as a potential conflict of interest
